# Psychiatric Adverse Effects of treatement with Corticosteroids: A Tunisian case report

**DOI:** 10.1192/j.eurpsy.2023.2032

**Published:** 2023-07-19

**Authors:** N. Boussaid, F. Guermazi, W. Abid, R. Masmoudi, I. Feki, I. Baati, J. Masmoudi

**Affiliations:** 1 Department of psychiatry, Hedi Chaker-Hospital-sfax-Tunisia, Sfax, Tunisia

## Abstract

**Introduction:**

Corticosteroids are certainly an efficacious treatment for several inflammatory and immunologic disorders. However, their abuse can lead to dangerous consequences such as psychiatric complications. Physicians and Psychiatrists should cooperate to treat and prevent, if possible, the deleterious adverse psychiatric effects of corticosteroids.

**Objectives:**

to describe a patient whose psychotic symptoms occured within 2 weeks of starting corticosteroid abuse, to review the literature and to suggest treatment.

**Methods:**

To present a case of a female young patient suffering from corticosteroid-induced psychosis due to corticosteroid abuse and review case report data published during the past quarter-century on adverse corticosteroid-induced psychiatric effects.

**Results:**

The patient was investigated to exclude other causes of her psychosis and she was treated with chlorpromazine and Risperidone. Numerous cases investigating these psychiatric corticosteroid-induced symptoms were identified. Data on incidence, drug dose, onset of symptoms, course of illness and treatment were arranged.

**Conclusions:**

Corticosteroid abuse should be put in the spotlight especially for young Tunisian females desiring to look plump. This misjudged abuse may have sever psychiatric complications. Thus we should establish strategies of prevention and cure to these psychiatric complications

**Disclosure of Interest:**

None Declared

